# Selenomethionine alleviates OTA-induced kidney injury in broilers by modulating ferroptosis

**DOI:** 10.3389/fvets.2025.1651205

**Published:** 2025-08-18

**Authors:** Wenqi Tian, Ruiwen Fan, Shuqin Zhou, Tao Liu, Yongdong Wang, Yuhang Sun, Miao Long, Shuhua Yang, Peng Li

**Affiliations:** ^1^Key Laboratory of Zoonosis of Liaoning Province, College of Veterinary and Animal Science, Shenyang Agricultural University, Shenyang, China; ^2^Heilongjiang Agricultural Engineering Vocational College, Harbin, China

**Keywords:** ochratoxin a, selenomethionine, broiler chicken, kidney, ferroptosis

## Abstract

Mycotoxin contamination in food and feed poses a significant threat to human and animal health worldwide. OTA is a common mycotoxin. About 20–30% of global feed is contaminated with OTA, and the annual potential contamination amount exceeds 200 million tons, which has become a major problem of local feed safety. OTA shows strong nephrotoxicity and causes kidney damage in animals and humans. Growing evidence suggests that OTA-induced renal damage is closely associated with ferroptosis. Selenomethionine (SeMet), as the main chemical form of daily dietary selenium supplementation, has pharmacological properties such as anti-oxidation, anti-inflammatory, anti-mutagenic, anti-cancer, anti-viral and anti-bacterial, which can effectively inhibit the nephrotoxicity of OTA. This study aims to establish an OTA-induced broiler kidney injury model and implement selenium-containing methionine intervention. Utilizing transmission electron microscopy, qRT-PCR, and immunoblotting techniques, the research will analyze pathological changes in broiler kidneys, examine ultrastructural alterations, and evaluate gene/protein expressions of renal function indicators, ferroptosis biomarkers, inflammatory-related parameters, and Nrf2-GPX4 signaling pathway components. This study aimed to investigate whether SeMet alleviates OTA-induced kidney injury in broilers by regulating ferroptosis and to elucidate the underlying mechanisms. Results showed that OTA caused renal histopathological alterations, elevated serum concentrations of urea nitrogen (BUN), creatinine (CRE), uric acid (UA), and pro-inflammatory cytokines (IL-1β and IL-6). OTA also increased reactive oxygen species (ROS), malondialdehyde (MDA), and ferrous ion (Fe^2+^) levels while reducing total antioxidant capacity (T-AOC), superoxide dismutase (SOD), and reduced glutathione/oxidized glutathione (GSH/GSSG). Additionally, OTA upregulated mRNA and protein levels of transferrin receptor 1 (TFR1) and kelch like ech-associated protein 1 (Keap1), while downregulating the nuclear factor erythroid 2-related factor 2 (Nrf2), heme oxygenase 1 (HO-1), quinone oxidoreductase-1 (NQO1), glutathione peroxidase 4 (GPX4), ferritin heavy chain 1 (FTH1), and solute carrier family 7 member 11 (SLC7A11), thereby inducing lipid peroxidation and ferroptosis. By reversing the above changes induced by OTA, SeMet alleviated OTA-induced renal injury and inhibited OTA-induced lipid peroxidation and ferroptosis. These findings indicate that SeMet alleviates OTA-induced renal injury by inhibiting ferroptosis, suggesting that SeMet can be used as a feed additive in mycotoxin-contaminated environments.

## 1 Introduction

Ochratoxins are a kind of metabolites produced by Aspergillus and Penicillium fungi, commonly found in mold-contaminated grains, feed, vegetables and fruits (1). Among ochratoxins (A, B, C, and D), ochratoxin A (OTA) exhibits the highest toxicity and poses significant risks to animal feed and agricultural products (2). Studies have shown that OTA has a variety of toxicological effects, such as teratogenicity, immunotoxicity, carcinogenicity, genotoxicity, and neurotoxicity, which may have an impact on health (3). The kidney serves as the primary organ affected by OTA, and evidence indicates that OTA may be the main pathogenic factor of endemic nephropathy in the Balkans (4). Oxidative stress is the primary focus of recent studies investigating OTA-induced nephrotoxicity (5). Nowadays natural antioxidants, such as curcumin, quercetin, catechin, and phytic acid, have been shown to mitigate OTA-induced renal damage (5–7). Nevertheless, additional mechanisms for mitigating OTA-related kidney damage require further investigation.

Ferroptosis, an iron-dependent cell death process, was marked by the accumulation of lipid peroxides, it was first identified in 2012 by Zhang et al. at Columbia University (8). In simple terms, ferroptosis is lipid peroxidation induced by iron accumulation, which eventually destroys the plasma membrane and leads to cell death. Ferroptosis has been implicated in mycotoxin-induced toxicity, as mycotoxins can destabilize iron homeostasis, impair antioxidant defenses, and promote lipid peroxidation (9). Recent studies increasingly highlight the critical involvement of ferroptosis in diverse renal pathologies, such as clear cell renal cancer, diabetes-related kidney disease, and AKI (acute kidney injury) (10–12). Although many of the disease associations in OTA are based on human studies, its toxicity to poultry is equally significant. High concentration of OTA in feed led to significant enlargement of chicken kidney, necrosis of renal tubular epithelial cells and deposition of urate (13). Oxidative stress is closely related to ferroptosis, iron-induced oxidative damage in cells catalyzes the synthesis of polyunsaturated fatty acids (PUFAs) and oxidative lipid degradation, which are the key elements of ferroptosis (14). Notably, OTA induces renal injury in mice via ferroptosis, marked by elevated iron levels and oxidative stress (15). Nrf2 is a pivotal regulator of gene transcription that regulates glutathione (GSH) levels by promoting the expression of GPX4 and SLC7A11 genes, thereby removing ROS and lipid peroxidation, maintaining cell membrane integrity and preventing oxidative damage which ultimately inhibiting ferroptosis (16). Research indicates that activation of the Nrf2/GPX4 signaling axis effectively suppresses ferroptosis (17).

Selenium (Se), an indispensable trace element known for its antioxidative effects, improves animal health and alleviates mycotoxin toxicity when supplemented in contaminated feed. Under normal circumstances, dietary Se mainly contains two forms: organic selenium and inorganic selenium. Organic selenium has better bioavailability than inorganic selenium. SeMet, widely utilized in humans and animals, enhances antioxidant and anti-inflammatory capacities by promoting selenoprotein synthesis, thereby maintaining physiological homeostasis (18). Research indicates that SeMet and sodium selenite have protective effects on OTA-induced renal injury in mice, which may be related to the reduction of oxidative stress, up-regulation of antioxidant enzyme expression and reduction of apoptosis (19). Moreover, Se is recognized as a key regulator of ferroptosis (20). GPX4, a selenoprotein critical for ferroptosis suppression, directly inhibits lipid peroxide accumulation, and its synthesis is Se-dependent (21, 22). For instance, recent research indicates that selenium inhibits ferroptosis in mice through the activation of the Nrf2/GPX4 signaling pathway (23). These findings underscore SeMet’s multifunctional roles in antioxidant defense, anti-inflammatory response, and GPX4-mediated ferroptosis inhibition. However, whether SeMet alleviates OTA-induced renal injury via ferroptosis suppression remains unclear.

Therefore, the present study established an OTA-induced renal injury model in broilers and investigated the protective effects of SeMet. Utilizing transmission electron microscopy (TEM), qRT-PCR, and Western blotting, we analyzed renal histopathology, ultrastructural changes, renal function biomarkers, ferroptosis markers, and inflammatory cytokine expression. Our findings aim to elucidate the mechanism by which SeMet mitigates OTA-induced renal injury through ferroptosis inhibition, providing a theoretical foundation for the application of SeMet in broiler production to counteract OTA nephrotoxicity.

## 2 Materials and methods

### 2.1 Materials

Freeze-dried Aspergillus ochraceus powder was purchased from the Guangdong Microbial Culture Collection Center (CGMCC 3.3876, Guangzhou, China). According to the instructions, strain activation culture was carried out in CDM and PDA medium. Feed was treated with a conidial suspension of Aspergillus ochraceus and maintained at 29–30°C for 14 days. OTA concentration was quantified using high-performance liquid chromatography with ultraviolet detection (HPLC-UVD) for subsequent experiments. Selenomethionine (SeMet) was procured from Zhejiang MinSheng Biotechnology Co, Ltd. (205,135 T, Zhejiang, China). The OTA dosage (1.0 mg/kg feed) was standardized at tenfold the maximum permissible threshold (100 μg/kg) defined by Chinese feed safety guidelines (GB 13078–2017) (24). The SeMet dosage (0.5 mg/kg feed) was based on previous studies demonstrating its efficacy in mitigating renal injury without adverse effects on broiler health (25). Therefore, 1.0 mg/kg OTA and 0.5 mg/kg SeMet were selected as the experimental doses.

### 2.2 Animal studies

All animal experiments were performed in compliance with the ethical protocols approved by Shenyang Agricultural University (No. 201806014) and followed the national legislative framework governing animal model research in China. The temperature of the chicken house was steadily maintained at 32°C (± 5°C) and the relative humidity was kept at 40% (±5%). During the feeding period, broilers had free access to feed and water, and a 12-h continuous light cycle was maintained. The broilers received a nutritionally balanced basal diet designed to comply with the National Research Council (NRC) guidelines for poultry growth.

One-day-old white-feathered broilers were purchased from the original chicken farm company of Shenyang Agricultural University. Sixty 1-day-old healthy white feather broilers were randomly allocated into four experimental groups after 1 week of adaptive feeding, with 15 broiler chicken in each group: Control group: maintained on a basal diet, OTA group: the diet was supplemented with 1.0 mg/kg OTA, SeMet group: the diet was supplemented with 0.5 mg/kg SeMet and OTA + SeMet group: the diet was supplemented with 1.0 mg/kg OTA and 0.5 mg/kg SeMet. The broiler chicken’ mental status and body weight were recorded every day for 21 days. After the last feeding, the broilers’ fasting body weight was measured without food and water for 24 h. Blood specimens were collected via cardiac puncture, and broilers were euthanized by cervical dislocation, and their kidneys were harvested for further experiments.

### 2.3 Hematoxylin–eosin staining

The tissue samples underwent immersion fixation in 4% paraformaldehyde (PFA; Sinopharm Chemical, Shanghai) followed by paraffin embedding. Serial sections of 5 μm thickness were prepared using a microtome, subjected to ethanol gradient hydration, and xylene-based dewaxing (2 h). Histochemical staining was performed with hematoxylin (Merck, Darmstadt) and eosin (Sigma-Aldrich), with subsequent dehydration through a graded ethanol series and xylene clearing. Permanent mounting was achieved using neutral resin. Morphological evaluation of renal tissue sections was conducted under bright-field illumination using a Leica DM750 microscope (Leica Microsystems, Beijing).

### 2.4 Transmission electron microscope observation

After the slaughter of broilers, samples should be taken within 1–3 min, a petri dish containing electron microscope fixative solution (2.5% glutaraldehyde) was prepared in advance before the sample. The small renal tissue blocks were removed from the body and immediately put into the culture dish, then cut into small pieces with a scalpel in the fixed liquid of the culture dish. The volume of the sampled tissue was controlled in the following sizes (1 mm × 1 mm × 1 mm cube, 1 mm × 1 mm × 3 mm rectangle, 1 mm × 2 mm × 3 mm flake). Mechanical damage such as squeezing of tweezers should be avoided, and the blade should be sharp to avoid contusion tissue. After the tissue was removed, it was put into the electron microscope fixation liquid immediately at room temperature and fixed without the light for 2 h, then transferred to 4°C preservation and transportation. The follow-up test was completed by Wuhan Sevier Biotechnology Co., Ltd., and the fixation liquid should not be frozen during storage and transportation.

### 2.5 Biochemical analysis

After blood collection, the blood was placed at room temperature for 2 h, centrifuged at 4000 rpm for 10 min, and the supernatant was sucked out. Creatinine (CRE), urea nitrogen (BUN), uric acid (UA), malondialdehyde (MDA), reduced glutathione/oxidized glutathione (GSH/GSSG), total antioxidant capacity (T-AOC), superoxide dismutase (SOD), reactive oxygen species (ROS) and ferrous ion (Fe^2+^) were conducted by biochemical kits from Nanjing Jiancheng Bioengineering Institute.

### 2.6 ELISA kit detection

The levels of the cytokines interleukin-1β (IL-1β) and interleukin-6 (IL-6) in serum were measured by ELISA kit (Shanghai Enzyme-linked Biotechnology Co., Ltd., Shanghai, China). All assay results were in line with the manufacturer’s recommended protocol.

### 2.7 qRT-PCR experiments

RNA from chicken kidney tissue was extracted by RNA extraction kit (Nanjing Noviacan Biotechnology Co., Ltd., Nanjing, China), and cDNA was obtained by reverse transcription of the extracted RNA by qRT-PCR kit (Nanjing Noviacan Biotechnology Co., Ltd., Nanjing, China). Specific primer sequences are listed in Supplementary Table S1 and quantified by SYBR green real-time PCR. This PCR was performed using a CFX ConnectTM real-time system (Bio-Rad, CA, United States) and AG™ SYBR Green Premix Pro Taq HS qPCR Kit (Accurate Biotechnology Co., Ltd., Hunan, China). qRT-PCR system: 2 × SYBRGREEN MIX 10 μL (Nanjing Noviacan Biotechnology Co., Ltd., Nanjing, China), upstream and downstream primers (10 μmol/L) 0.4 μL each, 2.0 μL of cDNA template, and 6.2 μL of ddH_2_O. We employed the 2^−ΔΔCt^ formula to assess mRNA expression levels.

### 2.8 Western blotting

A protein extraction kit (Solarbio Science & Technology, Beijing) was employed to isolate total protein from preserved renal tissue. The resulting lysates were then analyzed for protein concentration using the manufacturer’s BCA kit. Proteins were placed onto SDS-PAGE gels (Shanghai Yazyme Biotechnology Co, Ltd., Shanghai, China) and PVDF membranes were employed for protein transfer following electrophoresis. Membranes were blocked with 5% skim milk for 2 h and incubated overnight with their corresponding primary antibodies which were appropriately diluted: SLC7A11 (1:1000) (A2413, ABclonal Technology, United States), TFR1 (1:1000) (WL03500, Wanlei Technology, China), GPX4 (1:1000) (WL05406, Wanlei Technology, China), FTH1 (1:1000) (WL05360, Wanlei Technology, China), Nrf2 (1:1000) (WL02135, Wanlei Technology, China), NQO1 (1:1000) (WL04860, Wanlei Technology, China), Keap1 (1:1000) (WL03285, Wanlei Technology, China), HO-1 (1:1000) (WL02400, Wanlei Technology, China), and β-actin (1:1000) (YM8010, ImmunoWay, China). Subsequently, the membrane was incubated with enzyme-labeled secondary antibody (1:10000) (WLA023, Wanjiao, China) for 1 h at room temperature. Chemiluminescent substrates (Azure Biosystems, United States) were used to detect protein signals, with imaging performed on a chemiluminescence detection system. Quantitative analysis was performed using ImageJ (Version 1.46), with β-actin as the internal control. Target protein expression levels were normalized to β-actin OD values.

### 2.9 Statistical analysis of data

Data analysis was conducted with IBM SPSS Statistics 25 (SPSS Inc., Chicago, IL, United States), and outcomes were expressed as mean ± standard deviation (mean ± SD). For comparisons across experimental groups (control, OTA, SeMet, and OTA + SeMet), normalized data were subjected to one-way ANOVA. Graphical representations were generated via GraphPad Prism 8 (GraphPad Software, United States). Statistical significance thresholds were set at *p* < 0.05 (significant) and *p* < 0.01 (highly significant). Results surpassing these thresholds were deemed statistically reliable.

## 3 Results

### 3.1 Effect of OTA on body weight and organ coefficient of broilers and the protective effect of SeMet

**Figure 1:**
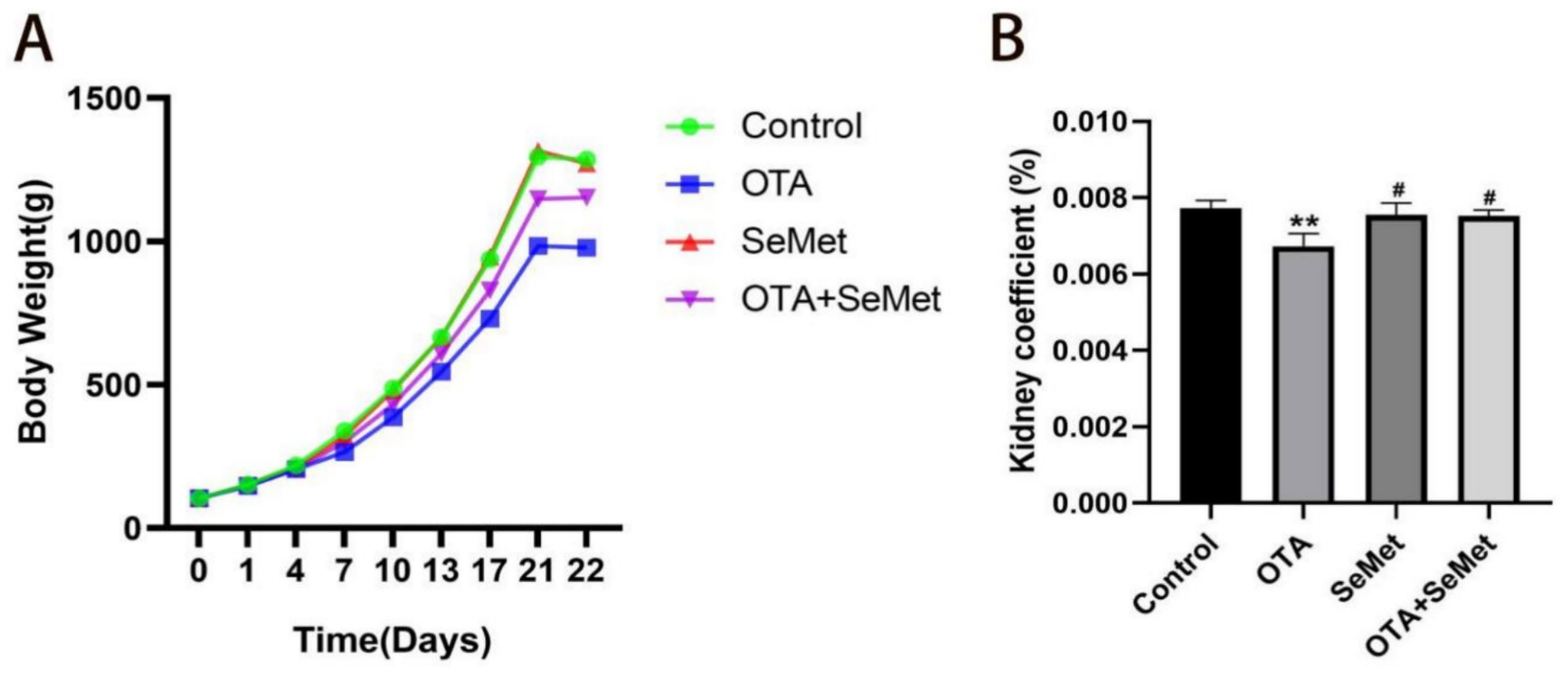
Effect of OTA on body weight and organ coefficient of broilers and the protective effect of SeMet. Results were reported as mean ± SD with six samples per experimental group. **(A)** Broiler weight growth change chart. **(B)** Broiler kidney index ratio. *: relative to control group, #: relative to OTA group. # represents statistical significance (*p* < 0.05). ** represents highly statistical significance (*p* < 0.01).

The weight and organ index of broilers in each group are shown in Figures 1A,B separately. Relative to the control group, the body weight and kidney coefficient of the broilers in the OTA group were substantially reduced (*p* < 0.01), while the changes in the SeMet group and OTA + SeMet group were not significant. At the same time, relative to the OTA group, the body weight and kidney coefficient of broilers in the OTA + SeMet group increased (*p* < 0.05).

### 3.2 Effect of OTA on renal pathological changes in broilers and the protective effect of SeMet

**Figure 2:**
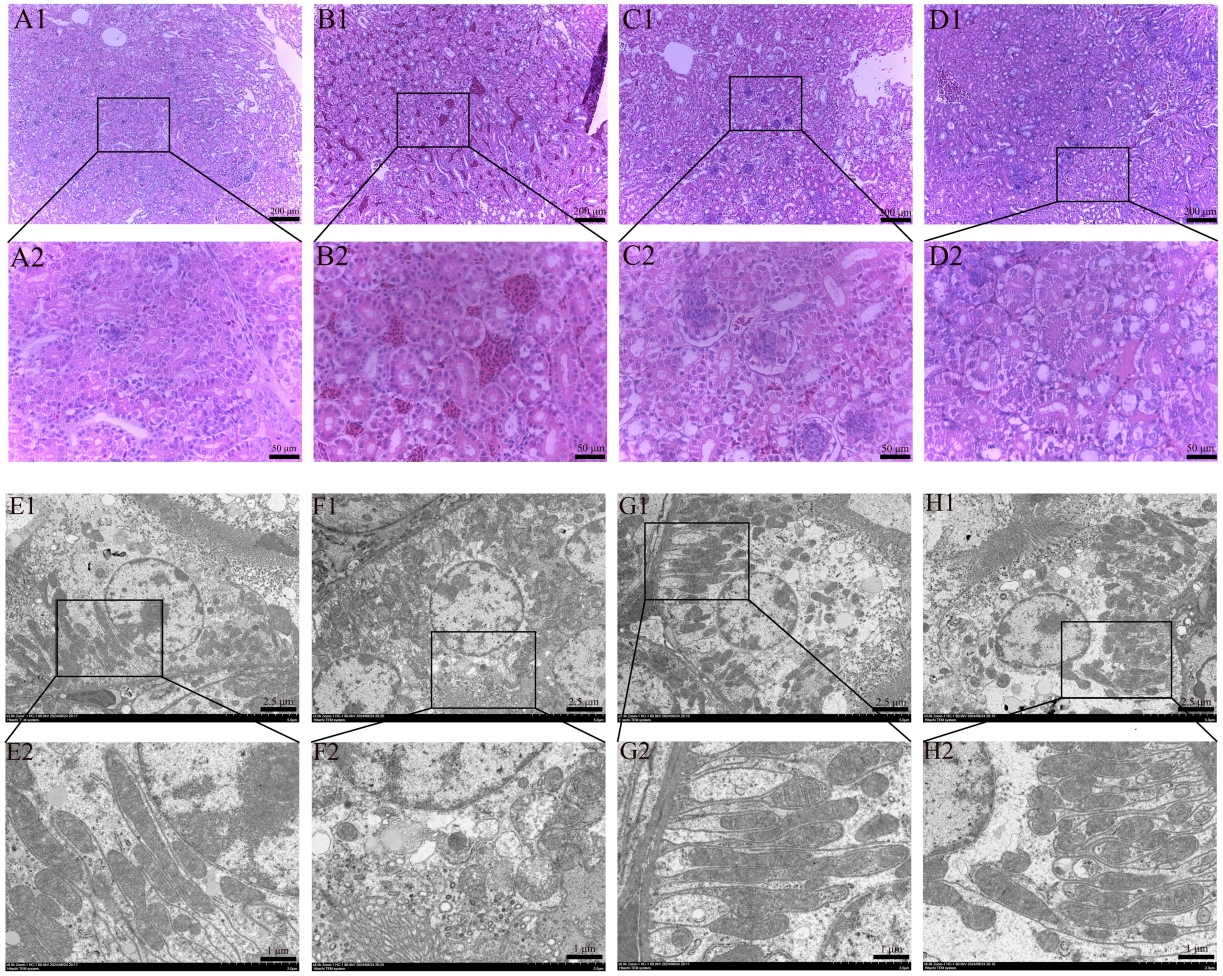
Effect of OTA on renal pathological changes in broilers and the protective effect of SeMet. **(A1,A2-D1,D2)** HE staining results of broiler kidney tissue. **(A1–D1)** Renal structure in 100-fold visual field. **(A2–D2)** Renal structure in 400-fold visual field. **(A1,A2)** Control group. **(B1,B2)** OTA group. **(C1,C2)** SeMet group. **(D1,D2)** OTA + SeMet group. Black arrows: inflammatory cell infiltration. Red arrows: Bleeding. **(E1,E2-H1,H2)** fluoroscopy results of broiler kidney tissue. **(E1–H1)** Electron microscopic results at 3000x field of view. **(E2–H2)** Electron microscopic results at 8000x field of view. **(E1,E2)** control group, **(F1,F2)** OTA group, **(G1,G2)** SeMet group, **(H1,H2)** OTA + SeMet group. Purple arrows: Outer mitochondrial membrane rupture. Yellow arrows: mitochondrial volume reduction. Green arrows: Mitochondrial ridges are reduced.

HE staining results of renal histopathological sections are shown in Figure 2, and the morphological structure of the kidneys in the control group (Figure 2A) and SeMet group (Figure 2C) was normal, and no significant pathological changes were observed. The glomeruli exhibited no structural abnormalities, the size of the renal sac cavity was normal and symmetrical, the tubular epithelial cells were arranged in a regular manner, the boundary was clear, the cytoplasm exhibited intense staining, and the nucleus was large and round. The distal convoluted lumen was large and clear, the nucleus was located on the surface of the proximal lumen, and the cytoplasmic staining was shallow. Relative to the control group, the OTA group (Figure 2B) showed a significant increase in the renal capsule cavity, renal tubular swelling, obvious inflammatory cell infiltration and bleeding. Relative to the OTA group, the space size of renal vesicles was substantially improved in the OTA + SeMet group (Figure 2D), and the inflammatory cell infiltration and hemorrhage were substantially restored.

The results of transmission electron microscopy were shown in Figure 2. The mitochondrial morphology of CON group (Figure 2E) and SeMet group (Figure 2G) was normal and complete. In the OTA group (Figure 2F), the mitochondrial volume decreased, the mitochondrial membrane ruptured, the membrane density condensed, and the mitochondrial ridge decreased, which was a typical feature of mitochondria when ferroptosis occurred in the cells. In the OTA + SeMet group, the mitochondria returned to normal and the morphology was relatively complete (Figure 2H).

### 3.3 Effect of OTA on kidney function in broilers and the protective effect of SeMet

**Figure 3:**
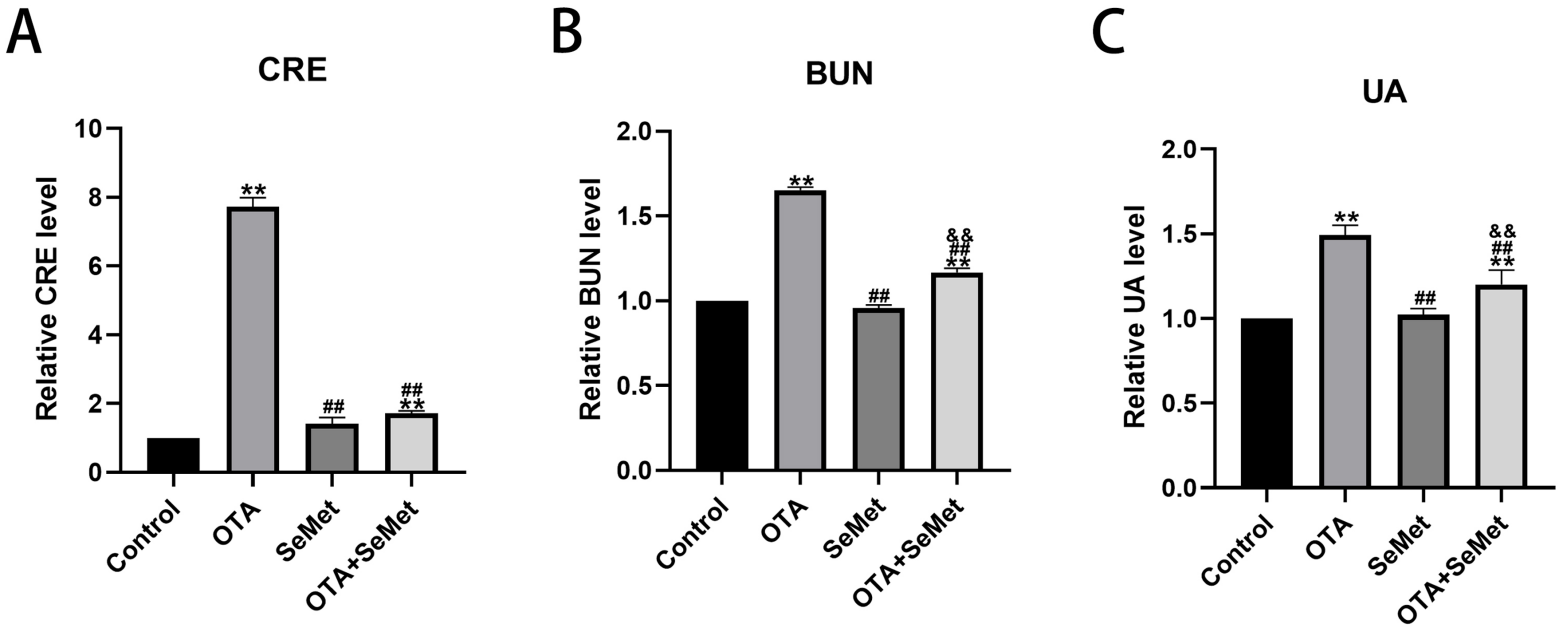
Effect of OTA on kidney function in broilers and the protective effect of SeMet. Results were reported as mean ± SD with six samples per experimental group. **(A)** Concentration of CRE in the serum. **(B)** Concentration of BUN in serum. **(C)** Concentration of UA in serum. *: relative to control group, #: relative to OTA group, &: relative to SeMet group. **, ##, && represents highly statistical significance (*p* < 0.01).

The renal function indexes of broilers in each group are shown in Figure 3. Relative to the control group, there was no significant change in renal function in the SeMet group. However, the serum concentration of CRE, BUN and UA in the OTA group and OTA + SeMet group were substantially increased (*p* < 0.01). Relative to the OTA group, the concentration of CRE, BUN and UA in the OTA + SeMet combined group were substantially lower (*p* < 0.01). Therefore, the data reveal that SeMet can alleviate the damage of OTA on kidney function in broilers.

### 3.4 Effect of OTA on renal inflammatory factors in broilers and the protective effect of SeMet

**Figure 4:**
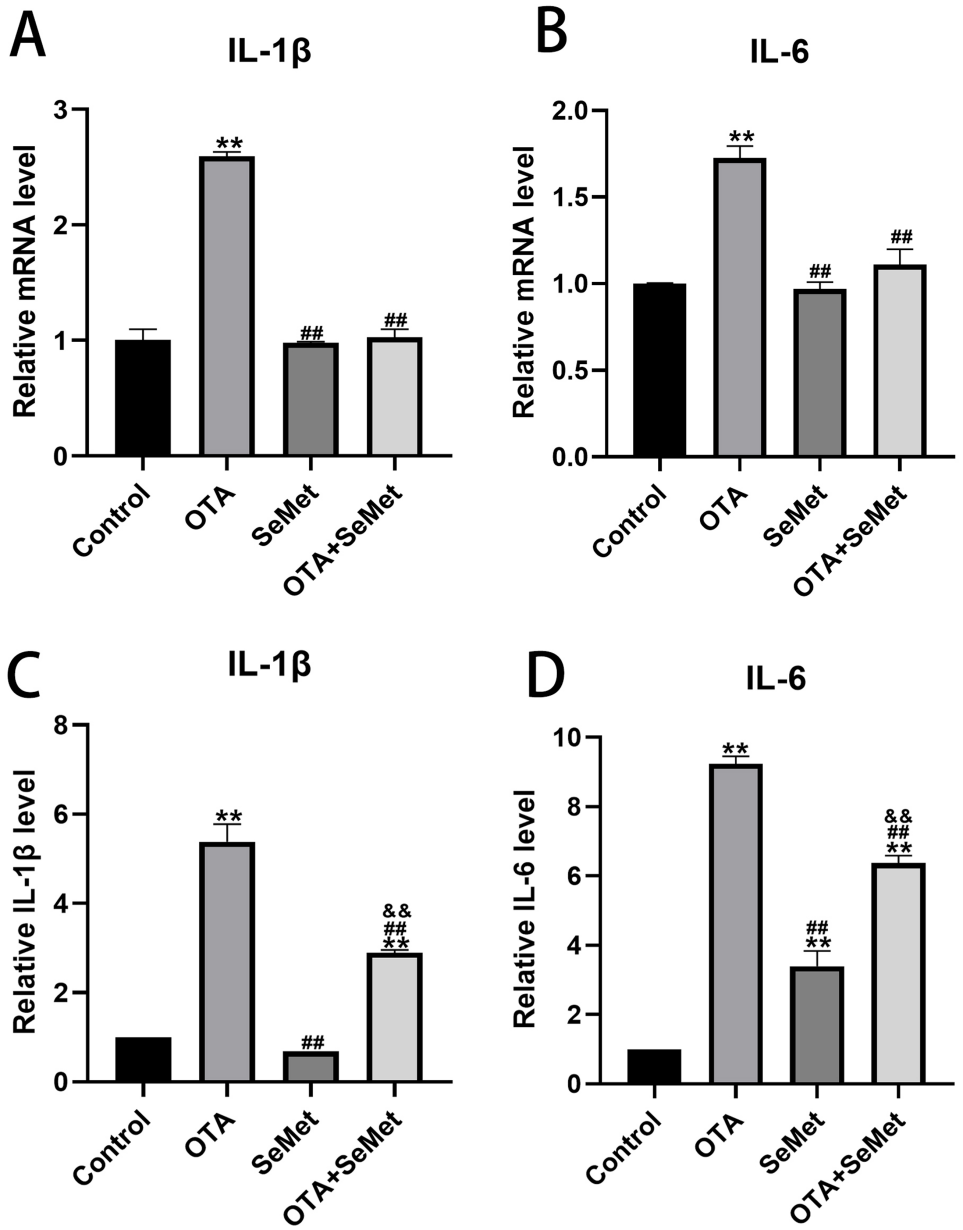
Effect of OTA on renal inflammatory factors in broilers and the protective effect of SeMet. Results were reported as mean ± SD with six samples per experimental group. **(A)** mRNA expression level of IL-1β in serum. **(B)** mRNA expression level of IL-6 in serum. **(C)** ELISA kit results for IL-1β in serum. **(D)** ELISA kit results for IL-6 in serum. *: relative to control group, #: relative to OTA group, &: relative to SeMet group. **, ##, && represents highly statistical significance (*p* < 0.01).

As shown in Figure 4, the qRT-PCR and ELISA test results of IL-1β and IL-6 in the kidney tissues of broilers in each group are shown. Relative to the control group, a significant upregulation of IL-1β and IL-6 was observed in the OTA group (*p* < 0.01), and the results of the SeMet group were comparable to those of the control group except for the ELISA kit detection results of IL-6, and the mRNA expression levels of IL-1β and IL-6 in the OTA + SeMet group were not substantially different from those in the control group. The concentrations of IL-1β and IL-6, as measured by ELISA, were substantially elevated compared to the control group (*p* < 0.01). Relative to the OTA group, the expression levels of the OTA + SeMet group were substantially reduced (*p* < 0.01). In summary, SeMet was able to attenuate OTA-induced increases in the expression of pro-inflammatory factors IL-1β and IL-6.

### 3.5 Effect of OTA on antioxidant indexes in broiler kidneys and the protective effect of SeMet

**Figure 5:**
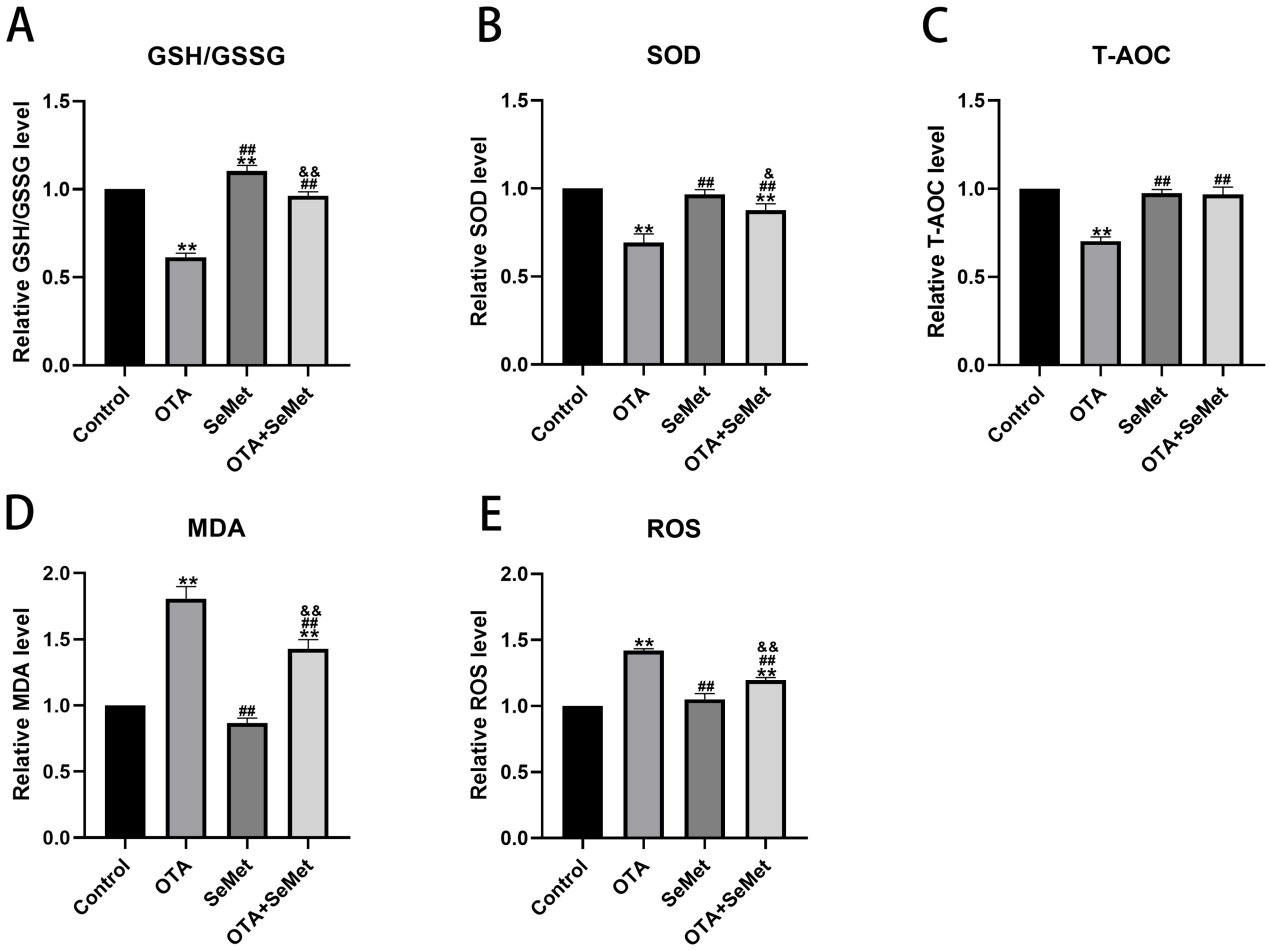
Effect of OTA on antioxidant indexes in broiler kidneys and the protective effect of SeMet. Results were reported as mean ± SD with six samples per experimental group. **(A)** Levels of GSH/GSSG in kidney tissue of broilers. **(B)** Levels of SOD in kidney tissue of broilers. **(C)** Level of T-AOC in kidney tissue of broilers. **(D)** Levels of MDA in kidney tissue of broilers. **(E)** Levels of ROS in kidney tissue of broilers. *: relative to control group, #: relative to OTA group, &: relative to SeMet group. & represents statistical significance (*p* < 0.05). **, ##, && represents highly statistical significance (*p* < 0.01).

As shown in Figure 5, relative to the control group, the levels of antioxidant enzymes SOD, T-AOC and GSSH/GSSG in the OTA group were substantially decreased (*p* < 0.01), while the levels of ROS and MDA were substantially increased (*p* < 0.01), and the levels of SOD, T-AOC, ROS and MDA in the SeMet group were not substantially different from those in the control group, and it is worth noting that the levels of GSH/GSSG in the SeMet group were substantially higher than those in the control group. Relative to the OTA group, the levels of SOD, T-AOC, and GSSH/GSSG in the OTA + SeMet group were substantially increased (*p* < 0.01), while the levels of ROS and MDA were substantially decreased (*p* < 0.01). In summary, SeMet was able to alleviate the generation of ROS, the accumulation of MDA, and the decrease in antioxidant enzyme activity caused by OTA.

### 3.6 OTA induces ferroptosis and SeMet protection in broiler kidneys by regulating iron metabolism and inhibiting Nrf2/GPX4 pathway

**Figure 6:**
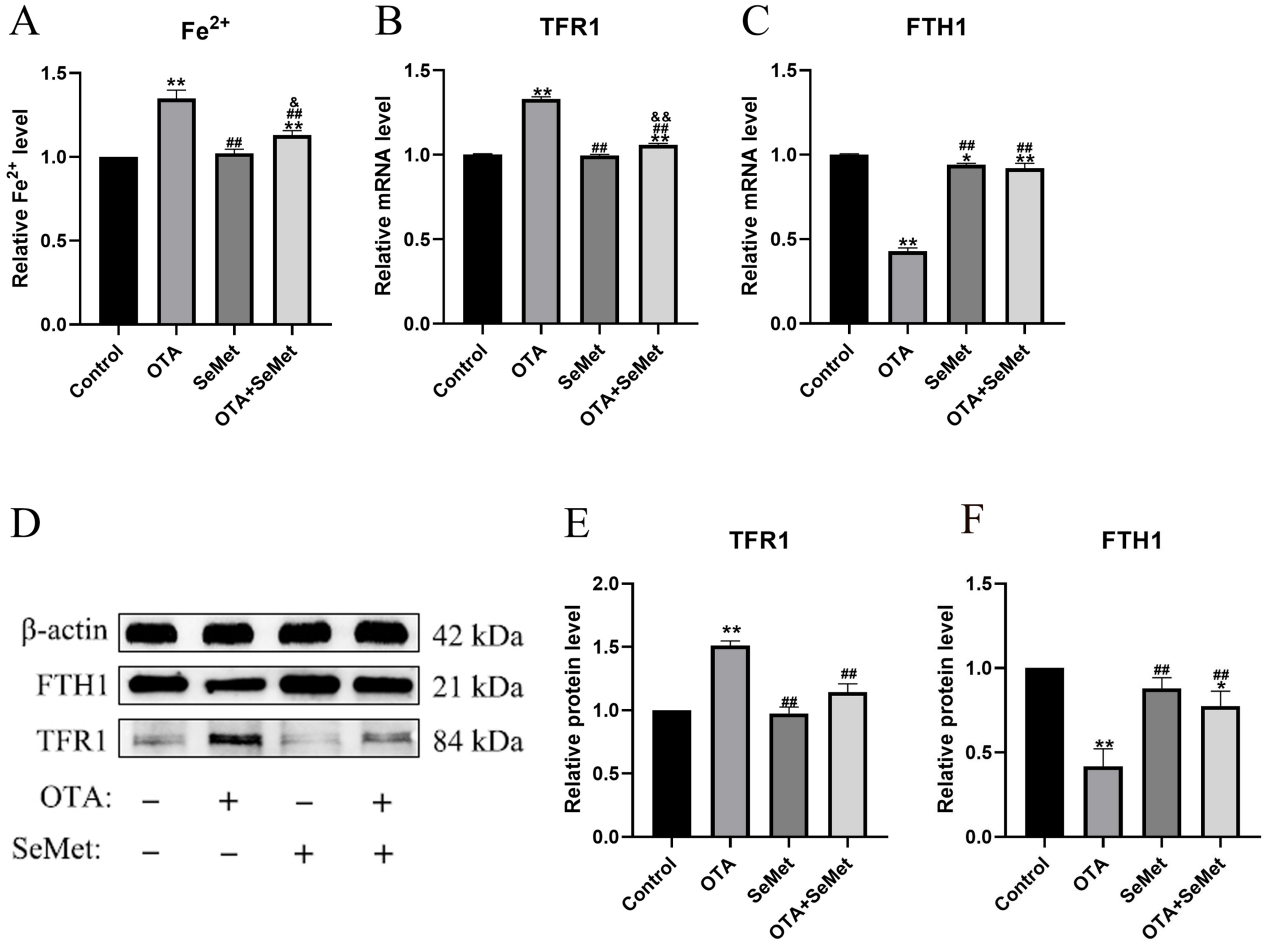
OTA induces ferroptosis in broiler kidneys by regulating iron metabolism and the protective effect of SeMet. Results were reported as mean ± SD with six samples per experimental group. **(A)** The content of Fe^2+^ in the kidney tissue of broilers. **(B–C)** mRNA expression levels of TFR1 and FTH1 in kidney tissue of broilers. **(D-F)** Protein expression levels of TFR1 and FTH1 in kidney tissue of broilers. *: relative to control group, #: relative to OTA group, &: relative to SeMet group. * and & represents statistical significance (*p* < 0.05). **, ##, && represents highly statistical significance (*p* < 0.01).

As shown in Figure 6, relative to the control group (*p* < 0.01), the ferrous ion content of the OTA group was substantially increased. In addition, OTA group substantially decreased the mRNA and protein expression levels of FTH1 and increased TFR1 (*p* < 0.01), and relative to the OTA group, the expression levels of FTH1 in the OTA + SeMet group were substantially increased (*p* < 0.01), while the expression levels of Fe^2+^ and TFR1 were substantially decreased (*p* < 0.01).

**Figure 7:**
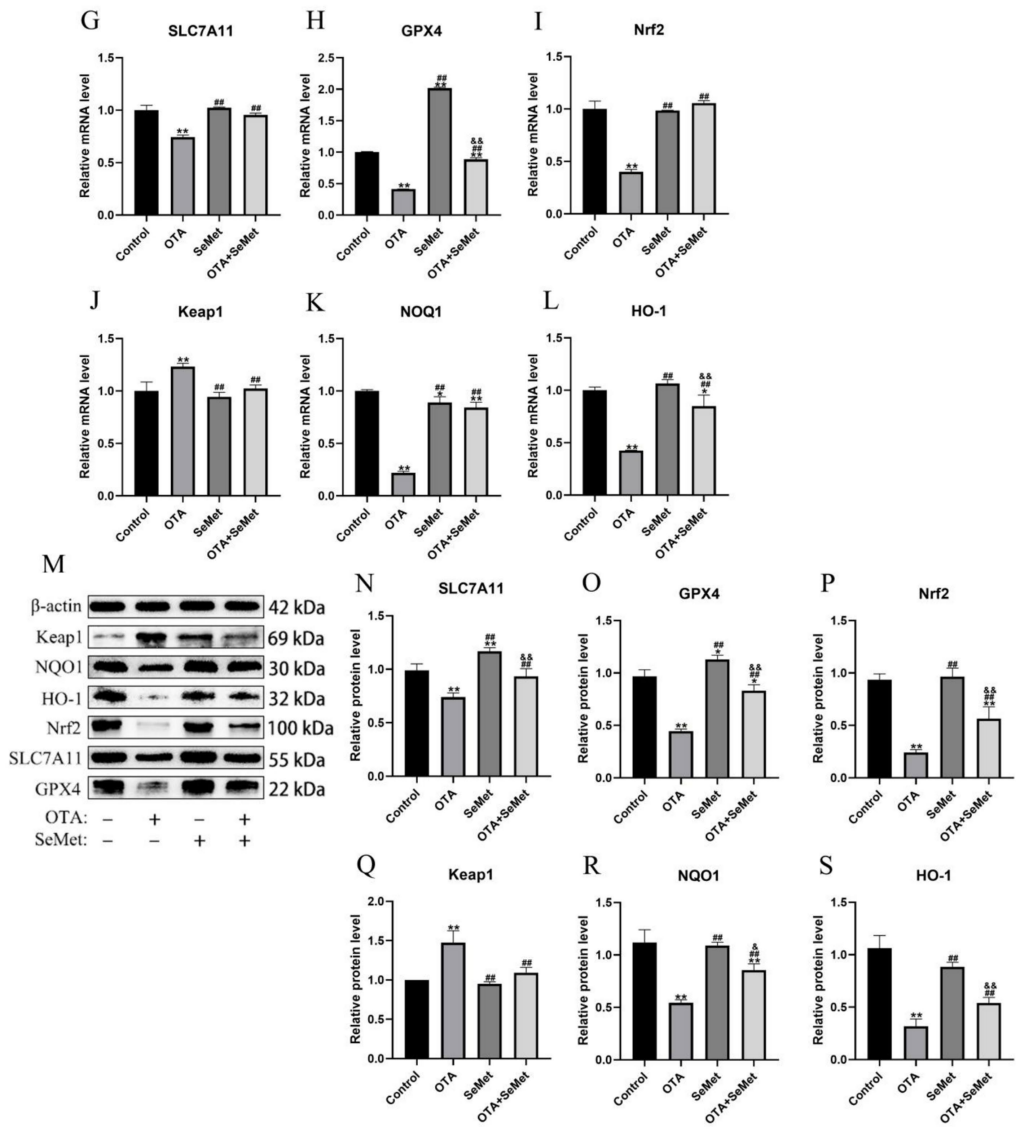
OTA induces ferroptosis and SeMet protection in broiler chickens by inhibiting the Nrf2/GPX4 pathway. Results were reported as mean ± SD with six samples per experimental group. **(G–L)** mRNA expression levels of SLC7A11, GPX4, Nrf2, Keap1, NQO1, and HO-1 in kidney tissue of broilers. **(M–S)** Protein expression levels of SLC7A11, GPX4, Nrf2, Keap1, NQO1, and HO-1 in kidney tissues of broilers. *: relative to control group, #: relative to OTA group, &: relative to SeMet group. * and & represents statistical significance (*p* < 0.05). **, ##, && represents highly statistical significance (*p* < 0.01).

As shown in Figure 7, relative to the control group, the mRNA and protein expression levels of Nrf2, GPX4, NQO1, HO-1 and SLC7A11 were substantially reduced in the OTA group (*p* < 0.01), and the mRNA and protein expression levels of Keap1 were increased (*p* < 0.01). In addition, we found that the mRNA and protein levels of GPX4 in the SeMet group were substantially higher than those in the control group (*p* < 0.05), indicating that SeMet substantially increased the level of GPX4. Relative to the OTA group, the expression levels of Nrf2, GPX4, NQO1, HO-1 and SLC7A11 in the OTA + SeMet group were substantially increased (*p* < 0.01), while the expression level of Keap1 was substantially decreased (*p* < 0.01). Our results suggest that OTA can induce ferroptosis by regulating iron metabolism and inhibiting the Nrf2/GPX4 pathway, while SeMet can inhibit ferroptosis by activating the Nrf2/GPX4 pathway.

## 4 Discussion

The kidney is the first target organ of OTA, and birds are the most sensitive to this toxin (26). After ingestion of OTA in animals, its concentration accumulation in the kidney is second only to that of blood (27). SeMet has been found to be an effective way to protect animals from OTA kidney injury (28). Therefore, we aimed to explore the role of SeMet in alleviating OTA-induced kidney injury in broilers. From the results of this research, it was known that relative to the control, SeMet group and OTA + SeMet group, the broilers in OTA intake group had the lowest body weight. Organ coefficient is an important indicator in toxicology to assess the effect of the drug (29), and it was also substantially reduced in the OTA group. Subsequently, it was further verified in histopathological observation, and it was seen that glomerular atrophy, significant inflammatory cell infiltration and hemorrhage occurred in the OTA group, while the pathological damage in the OTA + SeMet group was substantially reduced. In addition, SeMet substantially reduced serum CREA, BUN, and UA concentrations in the OTA group, consistent with previous studies (30). These results provide an experimental basis for SeMet to alleviate the renal damage of OTA.

Several studies conducted in a variety of animals have shown that the most prevalent health problem caused by OTA exposure is kidney inflammation (31, 32). OTA can make the organism to produce inflammatory factors by interfering with inflammatory signaling pathways, which in turn exacerbates the inflammatory response, simultaneously, it may directly influence immune cells, enhancing the secretion of pro-inflammatory mediators (33). Both the kit and qRT-PCR results used in the experiment showed that the levels of IL-1β and IL-6 were substantially increased in the group exposed to OTA, suggesting that OTA triggered inflammatory injury in avian renal tissues. However, after adding SeMet, the levels of IL-1β and IL-6 decreased in the combined group, this result is consistent with the findings of Zhao et al. (34). These results indicate that OTA-induced inflammatory response can lead to kidney damage, while SeMet has a protective role on OTA-induced kidney damage.

Several studies have revealed that inducing oxidative stress is one of the key mechanisms for OTA to exert its toxic effects. During OTA exposure, some of the oxidative defense enzymes involved in maintaining the balance of the oxidative defense system are disturbed. This study found that OTA led to a decrease in the levels of antioxidant enzymes such as SOD, T-AOC, GSH/GSG, and promoted the accumulation of MDA and the production of ROS, which is consistent with earlier research (35, 36). SeMet is a commonly used selenium supplement that exhibits significant antioxidant properties. In this experiment, Relative to the OTA group, the OTA + SeMet group increased the levels of SOD, T-AOC, GSH/GSG, while reducing the accumulation of MDA and the generation of ROS. These results indicated that SeMet had a certain protective effect on OTA-induced oxidative damage in kidney.

Ferroptosis is a novel type of cell death characterized by an imbalance of intracellular iron content and redox balance. An association between ferroptosis and kidney disease has been revealed, and strategies to suppress ferroptosis have been considered for the management and prophylactic measures for renal disorders (37). In this study, we found typical features of ferroptosis in chicken kidneys caused by OTA through electron microscopy, including mitochondrial atrophy and ridge reduction. At the same time, we found that OTA was able to induce ferroptosis by increasing the content of ferrous ions and lipid peroxidation levels in kidney tissue. Iron primarily exists in the organism as non-heme Fe^3+^ (ferric iron) and heme Fe^2+^ (ferrous iron) (38). TFR1 serves as a principal carrier protein that regulates intracellular iron levels by transporting Fe^3+^ into cells (39). Ferritin consists of two subunit types: FTL (light chain) and FTH1 (heavy chain 1). This protein complex plays a central role in cellular iron storage, binding the majority of intracellular Fe^3+^ (40). As a pivotal nuclear transcription factor, Nrf2 plays a central role in modulating cellular redox homeostasis (41). Under physiological conditions, cytoplasmic Nrf2 remains coupled with its negative regulator Keap1. Only upon phosphorylation can Nrf2 dissociate from Keap1 and translocate to the nucleus, thereby potentiating downstream gene expression (42). HO-1, a heme catabolizing enzyme, exerts antioxidant effects during cellular stress. Its expression is directly regulated by Nrf2 (14). Notably, GPX4, as the most important negative regulator of ferroptosis, is also a transcriptional regulatory target of Nrf2 (43). According to a study, the common sequence of ARE exists in the promoter of GPX4 protein, and GPX4 could be a target gene regulated by Nrf2 at the transcriptional level (44). Thus, the Nrf2/GPX4 axis is pivotal in inhibiting ferroptosis (45). The data from this research showed that OTA regulated iron metabolism and inhibited the Nrf2/GPX4 pathway to induce ferroptosis in broiler kidneys. The expression of TFR1 gene and protein in the OTA treatment group was substantially increased, while the levels of FTH1, SLC7A11 and GPX4 were substantially decreased. However, in the combined group, these changes were substantially alleviated. Selenium is an essential micronutrient that acts as an antioxidant stress. Selenium supplementation upregulates the expression of various selenoproteins, and the protective effect of the Se-GPX4 axis against ferroptosis has been demonstrated in follicular helper T cells (46). As a result, the level of GPX4 in the SeMet group was higher than in the other groups. SeMet has been shown to alleviate ferroptosis in the liver of mice caused by DON (deoxynivalenol) and restore the key proteins of ferroptosis, SLC11A7, GPX4, SLC3A2, and LPCAT3 to normal levels (47). The results of this experiment are consistent with it. In summary, this study demonstrates that SeMet effectively alleviates OTA-induced renal injury in broilers by inhibiting ferroptosis via the Nrf2/GPX4 pathway. It is recommended to supplement feed with SeMet (0.5 mg/kg) as a preventive measure in high-risk OTA contamination scenarios. However, further optimization of dosage and long-term safety assessments are required for practical application. Future research should explore the synergistic effects of SeMet with other antioxidants, investigate multi-organ protective mechanisms, and conduct field trials to develop comprehensive OTA mitigation strategies for the poultry industry.

## 5 Conclusion

Our study revealed that OTA can induce ferroptosis in broiler kidneys, which is manifested by the typical characteristics of ferroptosis in mitochondria and the elevated Fe^2+^ content in the kidneys on electron microscopy of the kidneys, decreased activity of antioxidant enzymes, increased activity of lipid peroxidation products, accumulation of ROS (reactive oxygen species), disorders of iron metabolism and inhibition of Nrf2/GPX4 pathway. On the other hand, SeMet can antagonize ferroptosis caused by OTA, thereby alleviating the damage caused by OTA to the kidneys of broilers. These findings provide new insights into the mechanisms of OTA-induced nephrotoxicity and open up new perspectives on OTA control and prevention strategies.

## Data Availability

The original contributions presented in the study are included in the article/Supplementary material, further inquiries can be directed to the corresponding authors.
