# Supplementary material for: Selenomethionine alleviates OTA-induced kidney injury in broilers by modulating ferroptosis
Source: Front Vet Sci. 2025 Aug 18;12:1651205. doi: 10.3389/fvets.2025.1651205 (PMC12400679; doi:10.3389/fvets.2025.1651205)

Supplementary Material

Supplementary Table S1 Primer pairs involved in RT-qPCR quantitative analysis in this study.

| **Gene name** | **Primer (5′–3′)** | **Sequence number**  **Primer size (bp)** |
| --- | --- | --- |
| β-actin | F:CCAGCCATGTATGTAGCCATCCAG  R:AACACCATCACCAGAGTCCATCAC | NM_205518.2  90 bp |
| IL-1β | F:CAGAAGAAGCCTCGCCTGGATTC  R:GCCTCCGCAGCAGTTTGGTC | NM_204524.2  127 bp |
| IL-6 | F:AAATCCCTCCTCGCCAATCT  R:CCCTCACGGTCTTCTCCATAAA | NM_204628.2  106 bp |
| SLC7A11 | F:GCTGTCGTGACGGTGCCTAATG  R:CTCTTGTGGCTGCCTGCTGTC | XM_040670527.2  87 bp |
| FTH1 | F:GAGGAGCGTGAACATGCTGAGAAG  R:ACACTCCATTGCAGTCAGTCCATTC | NM_205086.1  126 bp |
| GPX4 | F:CCGCTGTGGAAGTGGCTGAAG  R:ATCCTCCATTGGGCTGTACCTTTTC | NM_001346448.2  132 bp |
| TFR1 | F:AGGACCGCCTGTCATCTTCTGG  R:ACCTACCCTCCACCTCAAGTTGTC | NM_205256.2  97 bp |
| Nrf2 | F:GGGACGGTGACACAGGAACAAC  R:TCCACAGCGGGAAATCAGAAAGATC | NM_001396902.1  93 bp |
| NQO1 | F:CGAGTGCTTTGTCTACGAGATGGAG  R:AGGTCAGCCGCTTCAATCTTCTTC | NM_001277619.2  102 bp |
| Keap1 | F:GCATCACAGCAGCGTGGAGAG  R:GGCGTACAGCAGTCGGTTCAG | XM_025145847.1  150 bp |
| HO-1 | F:GCTGGGAAGGAGAGTGAGAGGAC  R:GCGACTGTGGTGGCGATGAAG | NM_205344.2  107 bp |

# Supplementary Figures


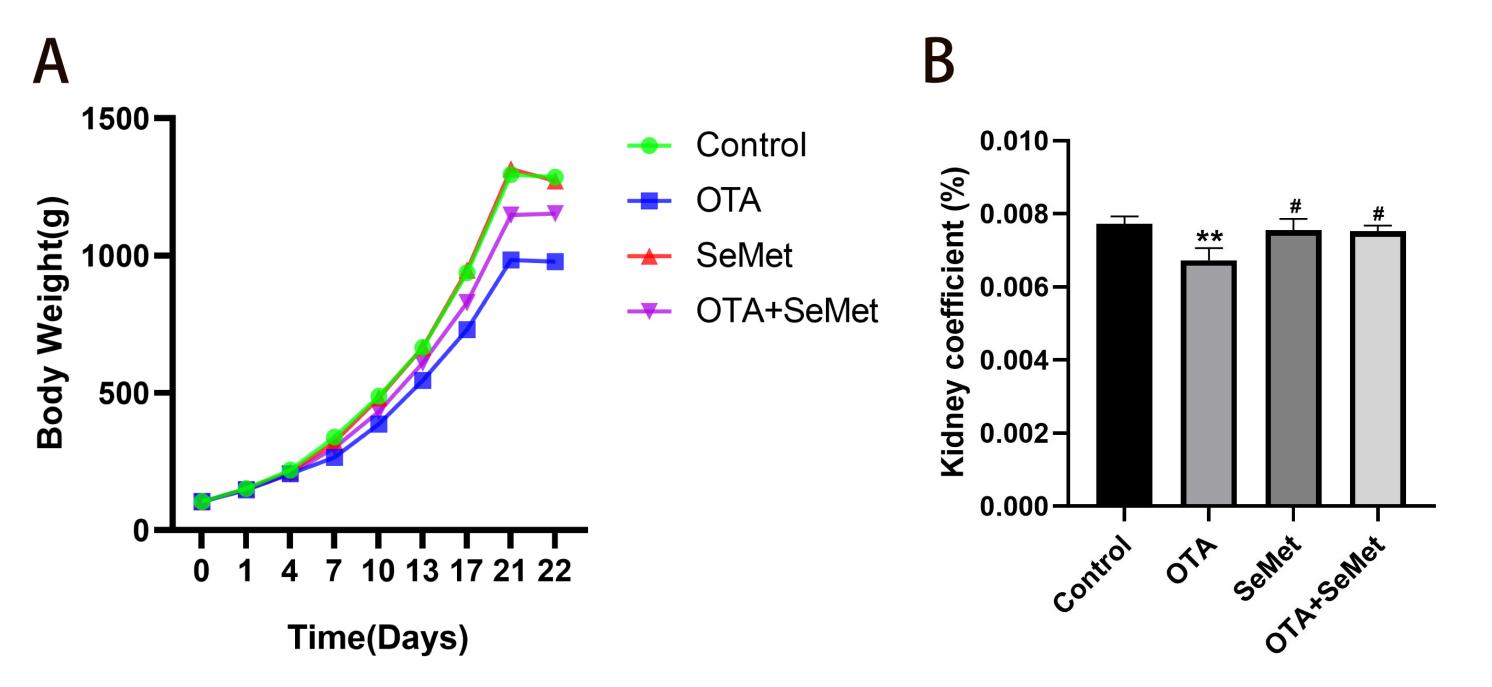


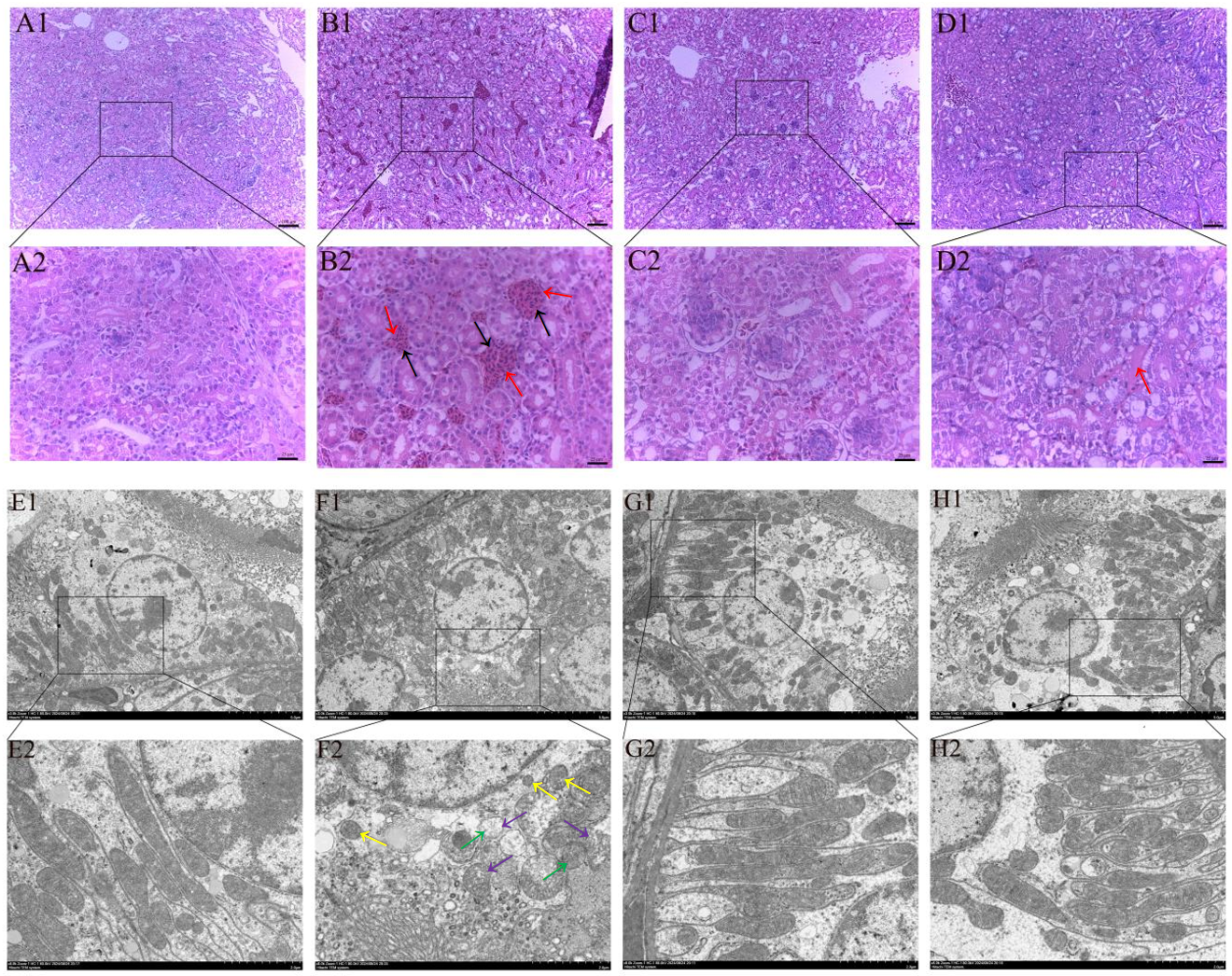


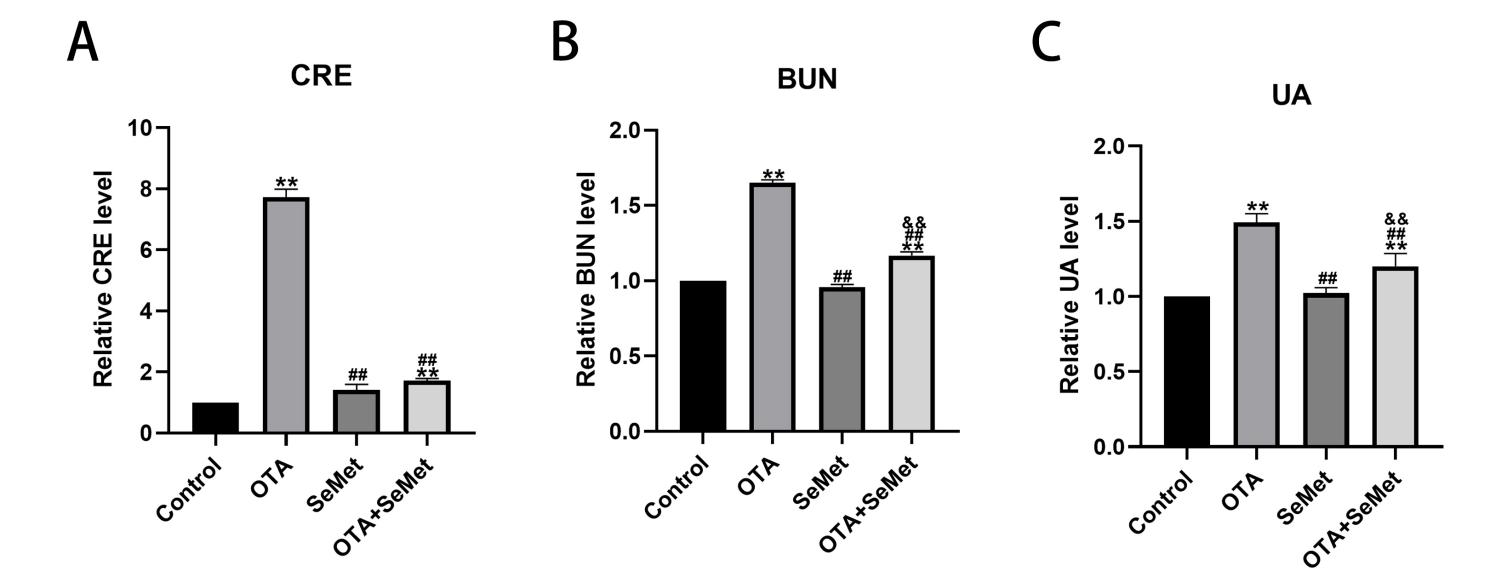


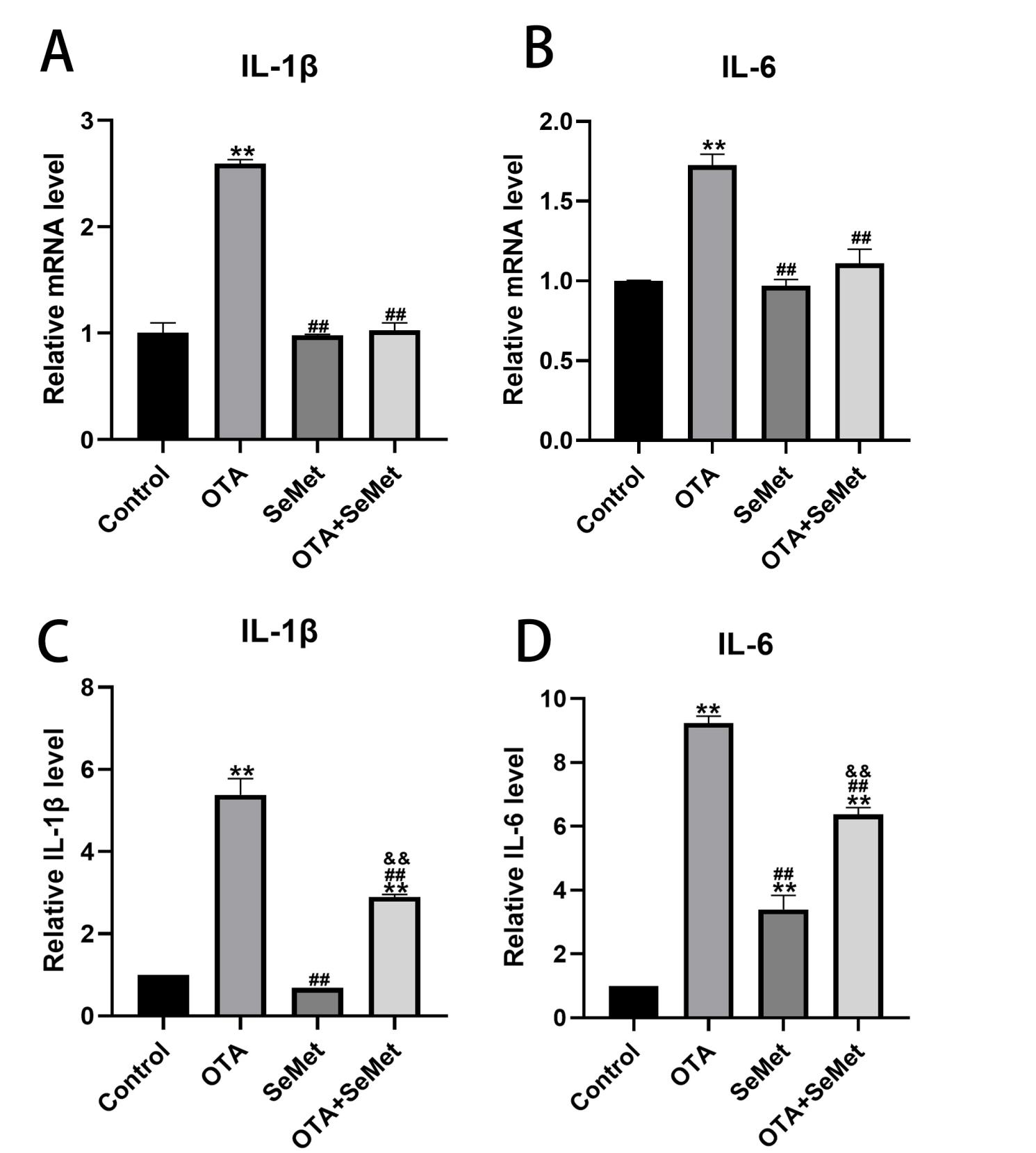


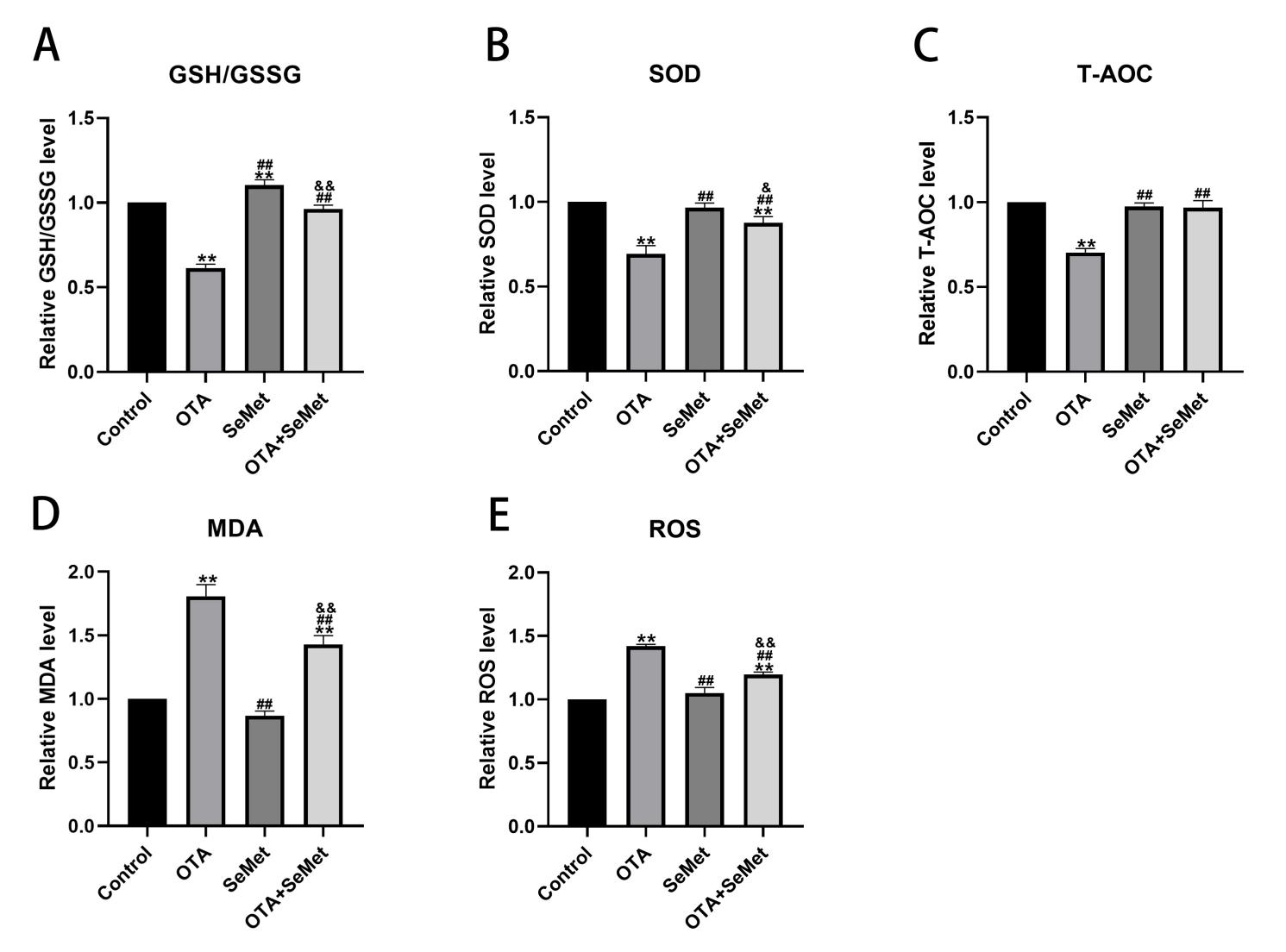


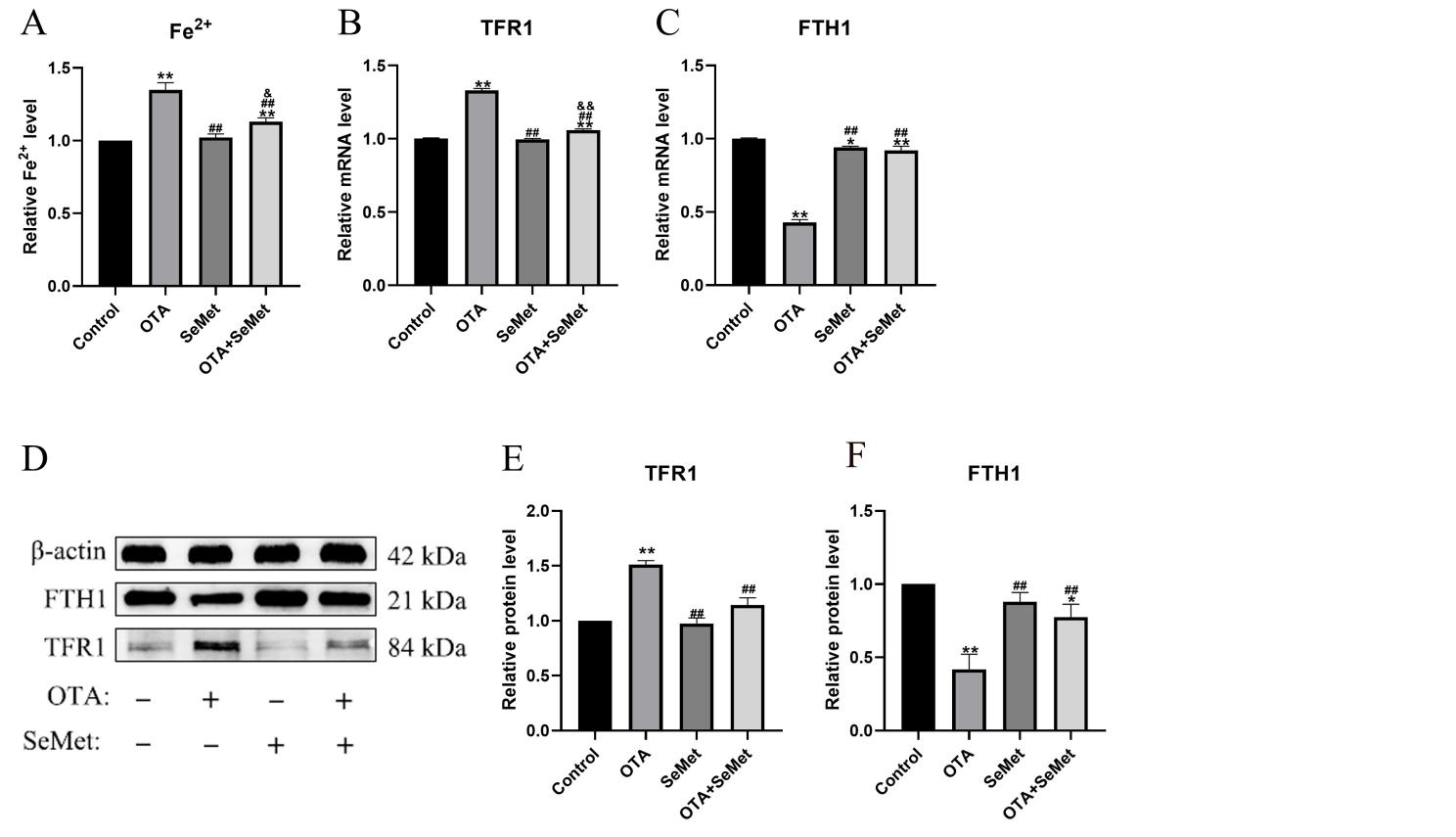


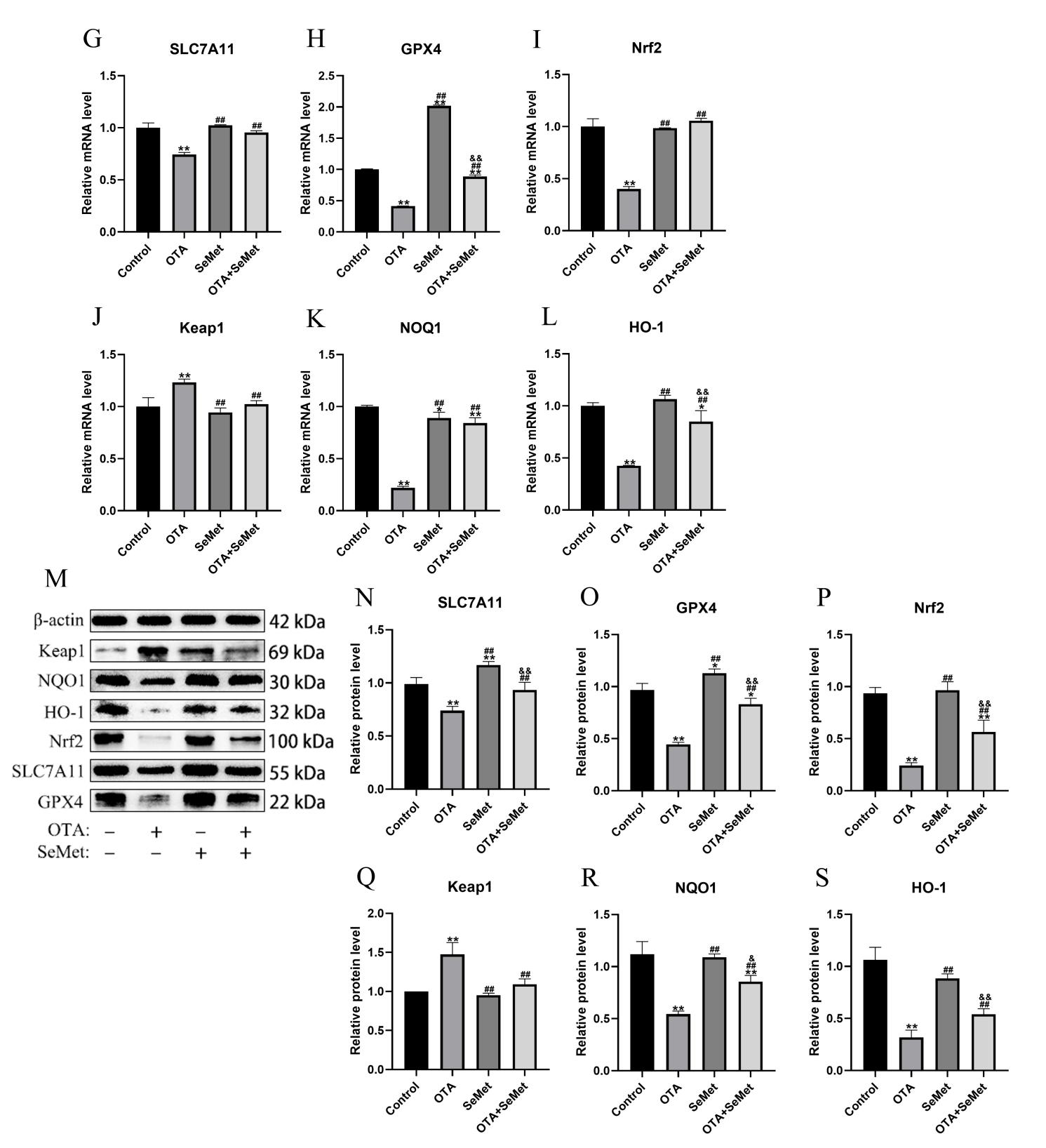

Supplement: Supplementary file 1 [file Table_1.docx]
